# The Staining of Tubercle Bacilli in Milk

**Published:** 1892-08

**Authors:** J. McFadyean

**Affiliations:** Royal Veterinary College, Edinburgh


### THE STAINING OF TUBERCLE BACILLI IN MILK.

By J. McFadyean, M.B., B.Sc.,
*Royal Veterinary College, Edinburgh.*

Thanks chiefly to the researches of Dr. Bang, of Copenhagen, the main features that distinguish tuberculous from other forms of mammary inflammation in the cow are now well known, and experience has shown that even without having recourse to a search for the tubercle bacillus in the milk, tuberculosis of the udder may, in many cases, perhaps in most, be diagnosed with tolerable certainty when the full history of the lesion is obtainable. Nevertheless, the absolute assurance that one has to do with a tuberculous mastitis is not obtainable except by demonstrating microscopically the bacilli in the milk, or by proving the presence of these organisms by inoculation or feeding experiments. When it is further taken into consideration that a diagnosis may have to be made in the case of an animal with no, or at least with no reliable, history, it is evident that the search for tubercle bacilli in milk has an important rôle to play in connection with the inspection of dairy cows.

As is probably known to most readers of this journal, the specific bacillus of tuberculosis is distinguished mainly by a peculiar staining reaction. The vegetative elements—cocci or bacilli—of most other species of bacteria are readily stained by simple watery solutions of the basic aniline dyes. The anthrax bacillus, for example, stains almost instantaneously with a watery solution of methylene-blue, gentian-violet, or fuchsin ; and, conversely, the stained bacilli are almost as readily robbed of their color by the action of comparatively dilute solutions of the mineral acids in water. On the other hand, the tubercle bacillus does not stain readily in the before-mentioned solutions. In order to stain tubercle bacilli with such dyes at ordinary temperatures, an exposure of twelve hours or more is required ; but when once the stain has penetrated, the organisms retain the color, even when exposed for a short time to 20 or 25 per cent. watery solutions of hydrochloric, nitric, or sulphuric acid. Fortunately, there are methods by which the penetration of the stain into tubercle bacilli may be greatly hastened. Thus by raising the temperature of the stain, or by adding to it ammonia or carbolic acid, the period required for staining can be reduced to a few minutes. The stain in most general use for tubercle bacilli at the present time is that prepared according to the Ziehl-Neelsen formula ; it is a solution of fuchsin in carbolized water (5 per cent.) with 10 per cent. of spirit added, and at a temperature of 60 degrees F. it may be relied upon to stain tubercle bacilli in ten minutes. The decolorizing agent employed in this case is a 25 per cent. solution of sulphuric acid in water. The essence of the method of staining may be briefly expressed by saying, that in a cover-glass preparation that has been stained for ten minutes in the Ziehl-Neelsen fluid, and then washed for a few seconds in the above acid solution, tubercle bacilli, and they alone, show a deep red color. It is true that the bacillus of leprosy has a staining reaction that is nearly identical with that of the tubercle bacillus, but for present purposes that is obviously of no importance.

It may now be considered whether there are any difficulties in the way of employing this stain to discover tubercle bacilli in milk. Several authors have recommended special formulæ for the staining of tubercle bacilli in milk, and it might, therefore, be assumed that the stain in common use for sputum and other ordinary materials had been found defective or unsatisfactory in the case of milk. Thus Arens in a recent number of the *Centralblatt für Bakteriologie* (Bd. XI, p. 9) recommends a chloroform methyl-blue stain for bacilli in milk and cream, while Scheurlen (*Ibid.* p. 54) for the same purpose fixes the cover-glass preparation by laying it for twenty-four hours in alcohol, and then, before staining, submits it for a day to the action of ether in order to remove the fat. The assumption that appears to underlie these special formulæ I believe to be erroneous. A cover-glass preparation made with either cream or milk containing tubercle bacilli may be stained quite successfully by the ordinary Ziehl-Neelsen method, and this statement is based upon numerous tests made with milk naturally containing tubercle bacilli, as well as with milk and cream to which tubercle bacilli from various sources were artificially added. I therefore feel justified in saying, that if any properly made cover-glass preparation of either milk or cream tubercle bacilli are not discoverable after staining by the Ziehl-Neelsen method, it may be assumed, with quite as much justification as in the case of a preparation of sputum similarly treated, that no such bacilli are present. In short, this difficulty with regard to the staining of tubercle bacilli in milk is an imaginary one.

There is, however, a real difficulty in the way of demonstrating tubercle bacilli in milk, and that is owing to the fact that even in fairly well advanced cases of mammary tuberculosis the bacilli may be very sparingly present in the milk. This, of course, is not a difficulty absolutely peculiar to the search for tubercle bacilli in milk, for it is present to a greater or less degree in the case of the other secretions and excretions in which the organisms are commonly found It is highly probable that tubercle bacilli are added to the milk only in those portions of the gland that are already the seat of a tuberculosis lesion ; and even although the bacilli are relatively abundant in the small amount of milk furnished by the diseased lobules, they are likely to be relatively few in the milk furnished by the whole gland, at least so long as any considerable part of the latter is not yet invaded by the disease. A few hundred bacilli would render a pint of milk highly dangerous, and yet a very laborious search, according to the common method, might fail to bring under view a single bacillus when they are present in such relatively small numbers. These reflections may serve to show how little importance ought to be attached to the negative result of an examination, even when several cover-glass preparations have been carefully searched.

There are two means by which, with the same amount of labor, the chances of discovering the bacilli may be considerably increased. The first of these consists in taking advantage of the natural tendency of the bacilli to gravitate to the bottom when a vessel of milk is set aside. With this object one may employ a burette as recommended by Mr. Coghill,* making the cover-glass preparation from the bottom stratum after the milk has stood for twenty-four hours. The same advantage may be obtained by allowing the milk to stand for that time in a six-inch test tube, and then drawing off for examination a little from the very bottom by means of a narrow glass tube used as a pipette. A still better apparatus, and one that can be extemporised at a small cost, is composed of an ordinary glass filter, to the lower end of which a piece of rubber tubing is attached, the latter being closed by a clamp. The funnel, supported in a wide-mouthed bottle or retort stand, is filled with the suspected milk, covered with a glass plate (or with a Petri's glass), and set aside for twenty-four hours. At the end of that time a little milk is run of from the bottom of the funnel by slackening the clamp on the caoutchouc tube. Although the bacilli tend to gravitate to the bottom of a vessel of milk, it is certain that some of them are carried up with the ascending milk globules and remain in the cream. It is, therefore, abvisable to examine preparations made from the latter also.

The second labor-saving modification is one that has not, so far as I am aware, been hitherto described. Ordinarily, films of milk, sputum, etc., are spread on cover-glasses, which cannot conveniently be used of a larger size than ⅞ of an inch in diameter. The quantity of milk that can be spread on such a glass is only the fraction of a drop, and it need hardly be said that the mechanical staining of half a dozen such preparations makes a serious demand on time Instead of making cover-glass preparations, I therefore make what may be called slide preparations; that is to say, I spread the film on a large microscopic glass slide (3 in. × 1½ in.), and, obviously, the amount of milk or other material that may be spread over one such piece of glass is equivalent to that which would make seven or eight ordinary cover-glass preparations. The film must not be made too thick, otherwise it is apt to peel off in the process of decolorising. When the film of milk has been uniformly spread over the surface of the slide with a glass rod, it is well to allow it to dry in an incubator kept at about 70 degrees F. If this is done it will be found that the film is both dried and fixed. The slide is then laid, film downwards, on a thin layer of the Ziehl-

* Woodhead's "Bacteria and their Products."

Neelsen stain in any flat glass vessel (a Petri's culture glass is very convenient) and left to stain for ten minutes. It is then decolorized in the ordinary way in 25 per cent. sulphuric acid solution, well washed in water, and dried. The entire film, equal to seven or eight cover-glass preparations, is now ready for microscopic examination. If this examination is made with an oil-immersion lens it is not necessary to apply a cover-glass to any part of it. It suffices to merely put a drop of the cedar or other oil on the spot to be examined, and proceed to focus the naked film. If a dry lens is used, it is well to place a drop of water on the part to be examined, and then put on a cover-glass. In either case it is obvious that the entire film is available for examination. If it is desired to make a permanent preparation of any part of the film in which bacilli have been found, all that is necessary is to wipe the oil or water from that spot with cigarette paper, and then put on a drop of Canada balsam and a cover-glass. Finally, the method is just as applicable to the examination of sputum, or scrapings from tissues, as to milk, and it admits of the use of a contrast stain when that is desired.—*The Journal of Comparative Pathology and Therapeutics.*

